# Coming of age: a qualitative study of adolescent girls’ menstrual preparedness in Palestinian refugee camps in the West Bank and Jordan

**DOI:** 10.1080/26410397.2022.2111793

**Published:** 2022-09-21

**Authors:** Rula Ghandour, Weeam Hammoudeh, Rita Giacaman, Gerd Holmboe-Ottesen, Heidi E. Fjeld

**Affiliations:** a Academic Researcher, Institute of Community and Public Health, Birzeit University, Said Khoury Building for Development Studies, P.O.Box 14, Birzeit, occupied Palestinian territory (oPt).; b PhD candidate, Department of Community Medicine and Global Health, Institute of Health and Society, University of Oslo, Blindern, PB 1130, 0318 Oslo, Norway; c Assistant Professor, Institute of Community and Public Health, Birzeit University, Birzeit, occupied Palestinian territory (oPt); d Professor, Institute of Community and Public Health, Birzeit University, Birzeit, occupied Palestinian territory (oPt); e Professor, Department of Community Medicine and Global Health, Institute of Health and Society, University of Oslo, Norway; f Associate Professor, Department of Community Medicine and Global Health, Institute of Health and Society, University of Oslo, Oslo, Norway

**Keywords:** Menarche, menstruation, menstrual preparedness, menstrual health, adolescents, Palestinian refugee camps, West Bank, Jordan, refugees

## Abstract

Menstrual health is important for adolescent girls and is particularly compromised in displaced communities due to restricted access to information and lack of private spaces to manage menstruation. Menarche is the biological and social milestone of girls’ adolescence, marking the onset of puberty and confirming womanhood in many communities. It also marks a difficult transitional period influenced by socio-cultural beliefs and expectations. Menstrual preparedness is critical for this transition, and the lack of accurate, timely, age-appropriate information might impact current, and future reproductive health and well-being. This paper investigates the menstrual preparedness status of adolescents living in Palestinian refugee camps in the West Bank and Jordan. These are long-term refugee camps characterised by a variety of social, economic, and political constraints affecting the health of women and girls. We conducted 39 in-depth interviews and 23 focus-group discussions with adolescent girls. The study reveals inadequate menstrual preparedness among the participants, especially in pre-menarche. Among the barriers to adequate menstrual preparedness is a predominance of practical concerns, such as the use of sanitary pads and hygienic practices, socio-cultural norms that promote secrecy and taboo around menstruation, and divergent notions of timeliness of information among girls, their mothers, and teachers. The study contends that addressing the taboo around menstruation requires joint efforts by the family, school, and social services. Menstrual preparedness should begin early and encompass biological, practical, emotional, and psychological components. The paper advocates for Comprehensive, Contextually Relevant, Timely Menstrual Preparedness (CCTMP) policies and initiatives, empowering adolescent girls, their mothers, and educators.

## Résumé

La santé menstruelle est importante pour les adolescentes, mais elle est particulièrement compromise dans les communautés déplacées, du fait d’un accès restreint aux informations et du manque d’espaces privés pour gérer la menstruation. La ménarche est l’étape biologique et sociale de l’adolescence chez les filles. Elle marque le début de la puberté et confirme la féminité dans beaucoup de communautés. Elle signale aussi une période transitionnelle difficile, influencée par les croyances et les attentes socioculturelles. La préparation menstruelle est capitale pour cette transition et le manque d’informations exactes, ponctuelles et adaptées à l’âge risque d’avoir des conséquences sur la santé sexuelle et reproductive et le bien-être futur. Cet article enquête sur le statut de préparation menstruelle des adolescentes vivant dans des camps de réfugiés palestiniens en Cisjordanie et en Jordanie. Il s’agit de camps de réfugiés de longue date, caractérisés par une variété de contraintes sociales, économiques et politiques qui touchent la santé des femmes et des filles. Nous avons mené 39 entretiens approfondis et 23 discussions par groupe d’intérêt avec des adolescentes. L’étude révèle une préparation menstruelle insuffisante chez les participantes, particulièrement avant la ménarche. Parmi les obstacles à une préparation menstruelle adaptée figurent la prédominance des préoccupations pratiques, comme l’utilisation de serviettes périodiques et de pratiques hygiéniques, des normes socioculturelles qui favorisent le secret et les tabous autour de la menstruation, et des notions divergentes sur la ponctualité des informations parmi les filles, leurs mères et les enseignants. L’étude affirme que pour s’attaquer aux tabous qui entourent la menstruation, des efforts conjoints doivent être déployés par les familles, l’école et les services sociaux. La préparation menstruelle devrait commencer tôt et englober les éléments biologiques, pratiques, émotionnels et psychologiques. L’article plaide pour des politiques et initiatives de préparation menstruelle ponctuelle, adaptée au contexte et globale, de façon à autonomiser les adolescentes, leurs mères et leurs éducateurs.

## Resumen

La salud menstrual es importante para las adolescentes y está particularmente comprometida en comunidades desplazadas debido al acceso restringido a información y a la falta de espacios privados para manejar la menstruación. Menarquia es el hito biológico y social de la adolescencia de las niñas; marca el inicio de la pubertad y confirma la femineidad en muchas comunidades. Además, marca un período transicional difícil influido por creencias y expectativas socioculturales. La preparación menstrual es crítica para esta transición, y la falta de información veraz, oportuna y adecuada para la edad podría afectar la salud reproductiva y el bienestar actuales y futuros. Este artículo investiga el estado de preparación menstrual de adolescentes que viven en campos de refugiados palestinos en Cisjordania y Jordania. Estos son campos a largo plazo caracterizados por una variedad de restricciones sociales, económicas y políticas que afectan la salud de las mujeres y niñas. Realizamos 39 entrevistas a profundidad y 23 discusiones en grupos focales con adolescentes. El estudio revela preparación menstrual inadecuada entre las participantes, especialmente antes de la menarquia. Entre las barreras a la preparación menstrual adecuada figuran: el predominio de preocupaciones prácticas, como el uso de toallas sanitarias y prácticas higiénicas, normas socioculturales que promueven secretismo y tabú en torno a la menstruación, y nociones divergentes del momento oportuno para recibir la información entre las niñas, sus madres y maestros. El estudio sostiene que para abordar el tabú relacionado con la menstruación se necesitan los esfuerzos conjuntos de la familia, la escuela y servicios sociales. La preparación menstrual debe iniciarse temprano y abarcar componentes biológicos, prácticos, emocionales y psicológicos. El artículo aboga por políticas e iniciativas de Preparación Menstrual Integral, Contextualmente Pertinente y Oportuna, para empoderar a las adolescentes, sus madres y docentes.

## Introduction

Over the last decades, there has been a growing awareness of the importance of menstrual health for improving women’s reproductive lives, including adolescent girls.^1,2^ Nevertheless, menstruation remained a relatively neglected issue in reproductive health research.^3^ Recently, we have seen renewed attention to menstrual health as a core global and reproductive health issue: for example, in two recent papers published in Sexual and Reproductive Health Matters by Wilson et al.^3^ and Hennegan et al.^4^ Menstrual health research has traditionally focused on Menstrual Hygiene Management (MHM) and Water, Sanitation, and Hygiene (WASH) programmes.^5^ However, more recently, there has been a stronger focus on menstrual preparedness, defined as “access to accurate, timely age-appropriate information”^4^ that takes into account broader socio-cultural factors.^4^

On average, girls reach menarche, i.e. the first menstrual event, between the ages of 12 and 13.^6^ Menarche is the key reproductive health milestone for adolescent girls, signalling the onset of puberty and, in many cultures, the transition to womanhood. This transition could have a variety of social consequences.^7^ According to studies conducted globally, and more notably in low- and middle-income countries (LMICs), including the Middle East, girls typically experience menarche with anxiety, fear, embarrassment, and perplexity.^7–9^ Furthermore, adolescent girls frequently lack access to adequate information about menstruation, its onset, implications, and self-care^7,8,10^; hence, they are unprepared.

Menstruation is closely embedded in socio-cultural norms, values, attitudes, and practices,^10^ where menarche and menstrual health discussions are often sensitive.^6,7,9^ Even in places where sexual and reproductive health issues are no longer taboo and are publicly discussed, there are numerous examples of women concealing menstrual indicators, indicating secrecy around menstruation as social etiquette.^11^

Healthcare services tend to address adolescents’ menstrual problems or irregularities as minor health issues, classifying adolescents overall as a healthy population subgroup.^12^ This is also reflected in reproductive health research, where menstrual health is peripheral and one of the least investigated topics.^8^ One example is the Guttmacher-Lancet Commission Report on “Sexual and Reproductive Health for All” from 2018, where menstrual health is mentioned only once and then described as an issue of managing menstruation in “a hygienic way, in privacy and with dignity”.^13^ Wilson and colleagues have recently argued for a need to integrate menstrual health more strongly within the sexual and reproductive health rights (SRHR) agenda.^3^ Moreover, experiencing barriers to achieving menstrual health can be described as “being denied basic human rights”.^14^ There is a global call to prioritise menstrual health as part of SRHR and human rights agendas. Importantly, this call argues for holistic approaches that include, but are not limited to, access to water, sanitation, and hygiene (WASH) facilities, as well as information and supportive social environments, addressing stigma and taboo linked to menstruation.^14,15^

## Background

### Menstrual preparedness

Being prepared for menstruation is critical for adolescent girls. Girls should be provided with accurate and timely information and awareness of menstruation before their first menstrual experience.^16^ Inadequate preparedness has been associated with negative emotions, reactions, and consequences. Lack of awareness about the changes occurring in the body and secrecy around menarche frequently result in experiences of body shame. Body shame has a detrimental effect on girls’ self-esteem and ability to cope with changes in bodily functions.^17,18^ It can compromise adolescent girls’ future abilities to negotiate their reproductive health choices, including sexual and fertility preferences.^3^

Menstrual preparedness research dates back to periods of intense debates in the 1970s and 1980s. In an insightful paper published in 1995, Koff & Rierdan identified three main aspects of menstrual preparedness that we still find applicable and relevant. First, girls need to learn and have access to information about the biology of menstruation and the practicalities of menstrual hygiene, including using and disposing of sanitary pads. Second, girls should be provided with emotional support, including a supportive environment; that is, privacy should be respected and teasing avoided. The third aspect relates to the psychosocial effect and meaning of menstruation, which is strongly linked to socio-cultural norms, values, and practices of menstruation.^19^ Adding to these three aspects, Wilson et al. noted that information about menstruation needs to be provided gradually and continuously, considering the girls’ age, developmental stage and needs, and socio-cultural norms and values.^3^ Hence, menstrual preparedness requires that information is accurate, timely, and age-appropriate.^4^

Although menstrual preparedness research has been less prominent over the past two decades, preparedness is often an integral part of other menstrual health agendas. For instance, the menstrual health and hygiene (MHH) approach and interventions targeting “period poverty”, i.e. lack of access to menstrual products, sanitation facilities, and adequate and timely information, emphasise menstrual preparedness.^20–22^ Access to basic WASH facilities is a challenge in many resource-poor settings worldwide,^5,23^ where girls face substantial barriers to access private spaces, clear water, and sanitary products. In addition, lack of access to adequate and timely information adds to these barriers to achieving menstrual health.

### Menstrual health in the refugee camp settings: the case of Palestinian refugee camps

The difficulties associated with achieving good menstrual health in resource-poor and densely populated contexts are worsened among displaced populations, affecting over 30 million women and girls due to wars or disasters.^24^ Menstruation management is challenging when access to WASH facilities and hygiene items is limited, especially within refugee settings.^25^ In addition, there are limited venues in refugee camp settings for discussing sensitive issues, such as reproductive health and other topics associated with embarrassment and taboo.^2^ Menstrual health is mentioned insufficiently, if at all, in recommendations and guidelines that define programmes in refugee camps, and when they are available, they focus on WASH facilities. Hence, there is a lack of comprehensive and holistic approaches to addressing women and girls’ needs in such circumstances, frequently omitting education and staff training.^24^

The Palestinian refugee camps in the West Bank and Jordan, where this study is based, consist of families who were dispossessed and forcibly displaced from their homelands following the 1948 Arab-Israeli war and the establishment of Israel on more than 50% of Historic Palestine. Palestinian refugee camps were created to host more than 750,000 Palestinians who were displaced in this process. Today, these camps serve their needs in the occupied Palestinian territory (oPt) (the West Bank and Gaza Strip) and three adjacent countries: Jordan, Lebanon, and Syria. The United Nations Relief and Works Agency for Palestine refugees in the Near East (UNRWA) was established in late 1949 to provide health, education, and relief services for Palestinean refugees.^26^ There are more than 13 million Palestinian refugees worldwide, with approximately 6 million registered by the UNRWA, of whom one-third continue to live in refugee camps.^26^ This study is concerned with Palestinian refugees residing in refugee camps in the West Bank and Jordan. Overall, there are 19 Palestinian refugee camps in the West Bank and 10 in Jordan.^26^

**Figure 1.**
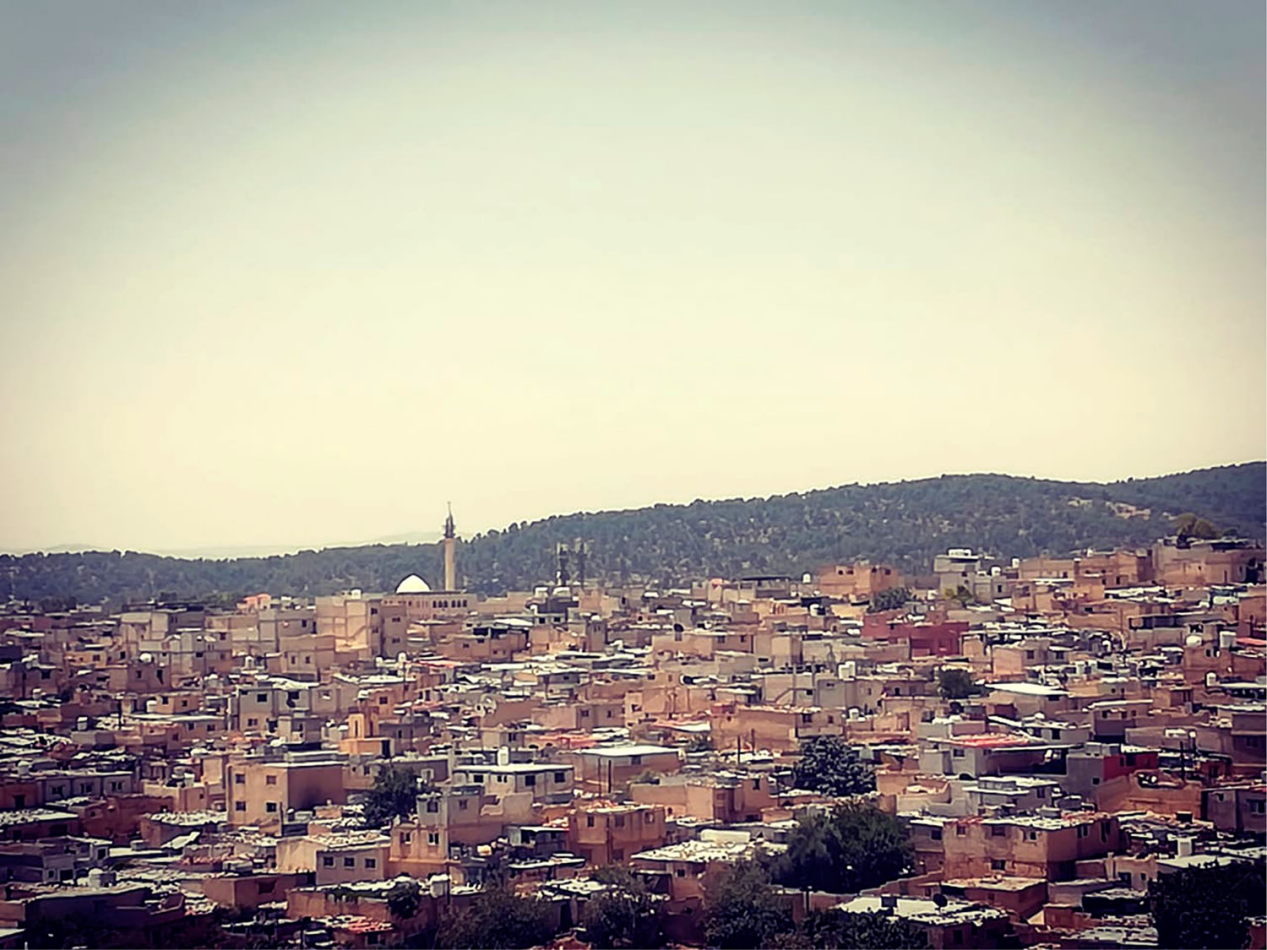
Al-Husun Palestinian Refugee camp in Jordan

Governance in Palestinean refugee camps is – until today – set with emergency response in mind,^27^ which is challenging as people living within these camps are long-term refugees. The long-term nature of their refugee status has transformed their needs for emergency support of food and shelter into more long-term problems of limited space and opportunities.^27^ Consistently, the refugees’ living conditions are worse for camp dwellers than for refugees outside camps.^28^ The areas rarely exceed one square kilometre per camp, and expansion beyond camp borders has never been possible.^26^ Thus, throughout the more than seven decades of camp life, expansion has only occurred vertically, leading to poor infrastructure, narrow alleyways, crowded households, and minimal privacy.^26,29^ The camps are very densely populated, with 30–40% of households having a crowding ratio of 3 persons or more per room.^28^ Today, the areas are more like urban slums than refugee camps (see Figure 1).

UNRWA has a somewhat unified services system across the camps, including for adolescents. Adolescent girls are eligible for basic education at UNRWA schools and health services from UNRWA health centres situated in the camp, free of charge.^28^ Available reproductive health research and programmes for women living in the camps target married women, and unmarried adolescents have minimal access to reproductive health services.^30^ A recent priority-setting study in the oPt found that educational institutions, service providers, research centres, and policymakers underestimate and underreport adolescent health needs, including reproductive health.^30^ This fact was further confirmed by a recent oPt scoping review addressing reproductive health research.^31^ Very little research has addressed menarche and menstrual health among adolescent girls in the West Bank and Jordan.^32–34^

This paper presents findings from a large-scale mixed methods research project on reproductive health needs among adolescent girls living in Palestinian refugee camps in the West Bank and Jordan. The paper focuses on menstrual preparedness as a core issue for achieving menstrual health. Employing qualitative research methods, we aim to better understand how girls were prepared for menstruation by examining the methods and sources of information used to inform them, as well as their reactions to the information and what they had learned. In addition, we aim to identify the main factors shaping and influencing their preparedness. We argue that menstrual preparedness for displaced Palestinian adolescent girls is jeopardised by several circumstances, starting from their home environment and extending to schools, health services, and the general community. Finally, the paper suggests a model that addresses menstrual preparedness at all levels.

## Methods

### Study approach

This paper draws on qualitative data from a larger study of adolescent girls in the Palestinian refugee camps in the West Bank and Jordan. The larger project aimed to address adolescent girls’ primary health information needs, including nutrition, anaemia, mental health, and reproductive health.^35^ The project consisted of three phases: an initial qualitative phase aimed to explore adolescent girls’ main health needs, a quantitative phase aimed to map and understand these health needs in a larger sample, and finally, an intervention phase aimed to identify ways to address and improve these needs. The present paper uses parts of the data from the first phase, exploring the main reproductive health challenges that emerged from the in-depth interviews (IDIs) and focus group discussions (FGDs) with adolescent girls, namely menarche and menstrual health.

### Participants

We recruited girls using local networks we had contact with in all refugee camps through purposeful sampling. We aimed for a maximum variation in age, school enrolment status, economic status, and camp locality. The majority of participating girls were 14–18 years old.* We conducted 23 FGDs with 193 participants and 39 individual IDIs with adolescent girls. The IDIs took place primarily at community centres, and the FGDs were conducted in community centres and schools (Appendix 1). All interviews and discussions were conducted in Arabic.

### Data collection

For data collection, we triangulated IDIs, FGDs, and observations. In the IDIs, we explored the girls’ personal experiences. In addition, the FGDs provided a setting for the girls to share experiences and to discuss community factors (cultural, social, and other) influencing their experiences. The interview guides for both the IDIs and FGDs asked about the girls’ first menstrual experience, sources of information before and after reaching menarche, knowledge, health-seeking behaviour, and if they have any specific need for more information about this topic (see Appendix 2 for the interview guides). The data collection was conducted by trained native Arabic-speaking female members of the research team who were also knowledgeable about the local socio-cultural context. Data collection was completed between March and September 2018. The IDIs and FGDs were audio-taped and transcribed in Arabic. In the FGDs, two or three trained research team members facilitated the discussion and wrote memos to document body language and other nonverbal communications. The IDIs and FGDs were conducted in enclosed spaces at the community centres or schools within camps to ensure girls’ privacy and confidentiality.

### Analysis

We conducted a thematic analysis to identify themes and subthemes, taking inspiration from Braun and Clarke^36^ but aiming to develop a more complex analysis connecting the themes to the broader socio-cultural and structural context. After familiarisation with the data and reading the transcripts several times, we developed a coding scheme. The first author coded data using the MAXQDA^®^ software. The research team continuously discussed codes, themes, and subthemes, developing initial analytical connections. Some transcripts were analysed by a different set of coders, and the Arabic-speaking authors checked the translations of identified quotes to enhance accuracy. Moreover, as a validity strategy, the first author, a Palestinian researcher who has relatives in refugee camps and is well informed about the situation in the camps, wrote self-reflective memos before and during the analysis and engaged in critical dialogue with the rest of the team.

### Ethical considerations

The study was approved by the Research Ethics Committee at Birzeit University (ref no. 171114) in December 2017 and by the Norwegian Centre for Research Data (ref no. 57983) in February 2018. Researchers and field workers obtained verbal consent from girls and their parents according to Birzeit University Research Ethics Guidelines. For FGDs at schools, approval from secondary school managers and principals was also obtained.

## Results

In total, 232 girls participated in this study with 193 girls taking part in the FGDs and 39 in IDIs. The girls’ ages mostly ranged between 14 and 19 years old. Two girls were above 19, but had menstrual experiences similar to those who were younger. Overall, in both the IDIs and FGDs, there were 54 girls who left school before completing high school, 13 girls had completed their secondary education successfully and 155 girls were enrolled in schools at the time of the study. Most of the participating girls were never married (222 girls), 5 were married and 5 were separated or divorced (see appendix 1 for details).

We present the results of this study in four main themes. The first theme is linked to timing and sources of menstrual preparedness. It describes how girls were prepared for menstruation, the persons involved in the preparedness, and when this happened. The second theme explores the content of preparedness (what was conveyed). The third theme reflects the main underlying factors that influence and shape the girls’ preparedness, including socio-cultural values and the refugee camp setting. The fourth theme sheds light on the strong link between menstrual preparedness and experiences.

### Timing and sources of menstrual preparedness

This theme describes the girls’ menstrual preparedness process. We found that menstrual preparedness was not timely, resulting in significant challenges for adolescent girls. The majority of the participating girls reported that they did not know about menstruation before its onset. Not surprisingly, the younger the age of menarche, the more this knowledge gap was evident. The girls’ narratives indicated that mothers tended not to discuss menstruation before the first period. One girl told us: “The mother will look at her daughter and say: no, not now, this is not your time”. The quote below from an adolescent girl who had her first menstruation in the fifth grade (around 11 years old) reflects this understanding among mothers:

*“I woke up crying and called my mother: I believe – there is something! My mother assured me that I should not be worried, and she promptly returned home from work. She told me: ‘You are blossoming [zahharti]’, which means you are a grown-up, and then she explained [about menstruation]. Then my menstruation stopped until seventh grade, when she [the mother] exclaimed, ‘I regret I had explained anything to you’.”*

The regret expressed points to mothers’ understanding that it is inappropriate to provide information on menstruation before menarche. It also shows that menarche is viewed as a positive event, conveyed as the “girl’s blossoming”. In most of the interviews, girls, especially those who had their first menstruation at a young age (less than 12 years old), reported that they learned about menstruation primarily at the menarchal event, and from their mothers.

Answering the question about where she learned about menstruation, one girl said:

*“They used to teach us this information at school as part of the health and environment curricula, and my mother used to inform me as well as my elder sister, who had her period earlier than me. And I gained knowledge.”*

Despite the multiple information resources that this quote mentions, most participating girls said they sought their mothers’ support at menarche. The mothers remained their primary source of menstrual information. Other female relatives, such as sisters, grandmothers, and sisters-in-law, were also involved, but to a lesser extent. Notably, health care services were rarely mentioned.

Many girls mentioned school teams as another key source of menstrual information and preparedness. For some girls, information about menstruation was part of their health education curricula between the seventh and ninth grades (13–15 years old). For others, the school health tutors or social counsellors would organise special extra-curricular sessions to discuss the topic. Less commonly, girls mentioned visitors from external institutions – such as university medical and nursing students or non-governmental organisations – coming to the schools to provide information about menstruation. It is worth noting that although most of the schools in the study area seem to address menstruation in some form, the discussions with girls indicated considerable variations in the effectiveness of conveying adequate information.

### Content of menstrual preparedness information

We found that information to enhance menstrual preparedness for adolescent girls strongly focused on hygiene and practical concerns and paid less attention to biological information, timely emotional support or the psychological meaning of menstruation. Although most girls reported that they had received adequate information on menstruation from their mothers upon menarche, when asked to specify the information provided, they mentioned practical aspects. These usually included menstrual hygiene, including using and disposing of pads and cleaning the body, as well as religious and social guidelines, including not fasting or praying while menstruating and keeping menstruation a secret. One girl said:

*“I got my first period in sixth grade. I went to my mother in tears. I felt both happy and frightened, and I was aware of what it was. My mother said, ‘I’ll bring you a sanitary pad.’ Naturally, I put it on incorrectly [laughs]. My mother placed it for me and informed me about it. She also asked me not to allow anyone to touch me, that my period would now occur every 30 days, and that ‘This is proof that you have matured’.”*

As with this example, mothers provided care in terms of practical advice, by pointing to some social consequences of menarche. Most of the girls conveyed that they felt emotionally supported by their mothers or close female relatives when they experienced menarche. Mothers tried to calm the girls down and normalise the event. This emotional support helped girls accept what was happening to their bodies and understand what to expect during their periods.

Yet, the girls’ accounts indicated that some information they received from their female relatives conflicted with what they learned from elsewhere. Some had been told by their mothers and grandmothers to avoid showering during their periods. When asked if showering is harmful during menstruation, one girl said:

*“My family is ignorant and holds erroneous beliefs. For instance, they discuss such things at home [that showering is harmful], but after we read a book assigned by the teacher, I returned home and informed them of the proper information. They were initially taken aback, asking, ‘What are you talking about?’ … My grandma said this to my mother, who relayed it to me. I inquired why I should abstain from showering during menstruation. Grandma stated: ‘If you shower when you first get your period, this is the end; there will be no childbearing, sexual organs contract, and then it [menstruation] ceases, and you are no longer menstruating.’ However, this proved to be incorrect – all chatter!”*

Very few girls reported sharing their grandmother and mother’s concerns about showering. On the contrary, many girls mentioned, as in the example above, that they were trying to rectify their mothers’ information with what they had learned from school, showing how proper education at school can empower girls and contribute to sharing information with female relatives.

Most girls lacked accurate knowledge about menstruation as a biological event. Many reported that they did not know why they menstruate, stating simply and directly: “I do not know”, or returned the question to the interviewer, asking: “Why do we have periods?”. Several girls thought menstruation had a cleansing effect, helping the body get rid of what was talked about as bad (*fased*) or impure (*najes*) blood, substances they perceived to have harmful effects. Menstruation, they believed, thus protected them from diseases. Highlighting the girls’ belief in the cleansing impact of menstruation, one girl said:

*“I am aware [of menstruation]. It absorbs sickness from the body. Consider my grandma; she is elderly and no longer menstruates, and as a result, she suffers from various diseases. For us, it safeguards our bodies against disease and removes poisons; this blood is bad, rotten and filthy.”*

Other girls believed menstruation was necessary for marriage and childbirth, but when questioned, they frequently could not explain how the two were related and produced vague and muddled responses:

*“It [menstruation] is for the girl when she gets married and such things. So I know each girl should have it, and it makes her feel psychologically better, and also, when she gets married, it is good to have a period.”*

Only a few girls gave a correct biological explanation of menstruation, explaining the ovulatory and uterine cycles and the fertilised ovum. However, these explanations were often incomplete and included both scientific and popular explanations, as this quote shows:

*“Yes, because the ovum was not fertilized, the blood should flow out, and the uterus will be destroyed, allowing it to rebuild. The blood that is oozing out is rotten (fased) blood.“*

Most girls maintained the notion that the blood had become bad and that, therefore, the body was getting rid of it.

The menstrual information from the schools, especially on the biological aspects, seems to be authoritative compared to that from mothers or other female relatives. However, due to the organisation of the curricula that directed instructors in their teaching of menstrual information in the schools, girls who left school at a very early age – before 14 years (the eighth grade) – received little information. For instance, biological information was included in the ninth and tenth-grade curricula, and preparedness material was included in the seventh-grade curricula in the West Bank but not in Jordan. This resulted in more extensive, yet not timely, menstrual information, in the West Bank.

### Underlying factors: socio-cultural norms and the camp setting

Talking openly about menstruation, especially in the pre-menarchal period, was considered inappropriate, shameful, and linked with embarrassment. To explain these emotions, the participants and other people in the camps used the term *aib* (“عيب ”*).* This poly-semantic Arabic word refers to something inappropriate, not acceptable, and impolite; something that leads to embarrassment and might be shameful or a taboo. That menstruation was considered *aib* to talk about was seen both in the individual interviews and even more strongly in the FGDs. The girls reacted to our questions with combinations of silence, giggling, murmuring, and smiling while looking at each other, and when they eventually started to talk, they lowered their voices, a cultural way of expressing embarrassment. With time though, the girls were more comfortable discussing their experiences in the FGDs, encouraged by the research team and more vocal peers.

Internalisation of *aib* informs and limits adolescent girls’ knowledge-seeking about menstruation. Many girls expressed a disinterest in learning about menstruation before its onset. When asked about menstruation, one girl who had not yet reached menarche said:

*“I do not know a lot; I am not used to talking about such issues and am not concerned. I do not like talking about such topics, and I feel embarrassed.”*

Many girls were also too embarrassed to ask the teacher for information: “*This is a topic I am embarrassed to talk about; I will never ask anybody*”, said another participant, reflecting the association of *aib* and menstruation for the adolescent girls. *Aib* was also significant in menstrual education at schools. Teachers, health tutors, and social counsellors were reluctant and embarrassed to discuss menstruation openly, according to the girls. One girl said: “*The teacher will say go ask your mothers”*. Furthermore, when school teams discussed these topics, the girls would giggle and laugh, making it difficult for teachers and counsellors to control and finish the lesson, and many times they would just cancel the class and ask the girls to seek knowledge by themselves elsewhere. One participant described her class thus: “*Because when teachers spoke to us [about menstruation], the girls would mock the subject, and the teacher would say enough and quit teaching the remainder of the session.”*

Teachers were also confronted with community opposition from some girls’ families to menstruation health education in school, which was also based on the notion of *aib*. One girl said:

*“When we were in the fifth grade, we asked our teacher to explain menstruation. ‘No, I did that last year, and one of the mothers came to school and scolded me for explaining this to girls who are still too young,’ she explained.”*

Openly discussing menstruation was also difficult in the private sphere, and *aib* is amplified by gender norms where it was inconceivable to discuss any sexual reproductive health issue openly in front of males and including close relatives. One girl told about the situation when she had her first period demonstrating the difficulty of approaching the subject of menstruation in the presence of males:

*“Oh God, I was in agony. I was in the sixth grade and on my way to school when I got my first period. They [the school] escorted me home. My mother was at home but unaware of what was going on [around her] because we were having a funeral. My uncle and cousin were in our building. That day was a public scandal.”*

The need for gender-segregated space to discuss such sensitive topics is difficult to meet in the Palestinian refugee camp settings. We found the camp setting impacted girls’ menstrual preparedness and experience in various ways, complicating and amplifying the effect of *aib*, thus adding to the challenges for adolescent girls to discuss menstruation within and between households and at school. The camps are crowded, and the small houses host extended families forming networks of close kin, making it hard to discuss sensitive issues openly. One girl said:

*“You will not feel at ease at home. The houses are connected; your neighbours will hear the conversations you conduct with your family. For instance, our neighbour overhears every word we say because our house is attached to theirs.”*

The structural conditions of the camp also influenced the possibility of observing basic hygienic practices. Although girls seem to have adequate information about using and disposing of sanitary pads, in practice this was extremely hard to do at the schools. Even though WASH facilities were available at all schools, their functionality was a major concern for girls, reporting that the toilets were usually dirty, with no tissue paper, soap or trash bins. Although running water was available, dirty sinks and floors were barriers to using this water. Girls said they used toilets at schools only in extreme cases. One girl said about using sink water at school:

*“You will never use the soap after anybody. The sinks are full of dirty water and clogged with tissue paper, and you will never think of washing your hands.”*

Not using the toilets for long hours had health risks that girls were aware of but could not help. As the schools were girls-only with primarily female teachers, the discussions did not bring out any gender issues related to WASH. However, the concealment and secrecy around using toilets during menstruation remained dominant as reported by participants. Given the small geographical space of the camps, the close proximity of their households to the school allowed the girls to go home to use toilet facilities, solving parts of the challenges related to accessing WASH facilities.

### Menstrual preparedness and girls’ menarchal experience

From the girls’ perspectives, menarche was a significant and unforgettable life event. Many girls could retell their first menstrual experience in much detail. The overarching finding is that menarche was a dramatic life event, reflected in the strong words many used to describe the situation, such as feeling “horrified” and experiencing a “black day”. Box 1 presents a narrative from one of the participating girls that exemplifies the many worries surrounding menstruation’s initial commencement.

Box 1. **A narrative given by an 18-year-old girl about her first menstruation experience (the story has been slightly edited for clarity after translation). Her narrative can serve as an example of several worries surrounding menstruation’s initial commencement.**

*I had my first period when I was about 12 years old. I was terrified the first time I got it. I was at school when I suddenly felt something, and I thought to myself, What just happened?! I requested permission from the teacher to use the restroom. I was gazing and thinking, “What is this?” while on the toilet. I began to cry, and I remained in there for an extended period of time. Naturally, the lesson ended while I was still in the restroom. My friends arrived. “What is the matter?”, they inquired. “Nothing,” I replied, “I need to see the principal”. I went over there and informed her. “It’s all right, my love; do not be afraid,” she stated. I asked, “What is this? I was curious to know. Of course, we all know a little about menstruation, but I had no idea it was this way.” “It’s all right,” the principal responded. She contacted my mother, who arrived. “Let us go home,” she said. I returned home, took a shower, and my mother explained to me: “When you have your period, no one is permitted to know; this is yours alone.” It is, for example, forbidden to discuss it with your friends or aunts … When I returned to school the following day, my friends inquired, “How come you left yesterday?”. What am I to say to them? I informed them that I was experiencing abdominal pain and hence returned home. Of course, I did not inform them that I had had my period, but they were aware, How?! Because my blood-soaked clothes stained the chair. So, I told them: ok, you know it is normal, but if you tell anybody else out this group, you would see what will happen to you. Yeah … It was quite difficult at first, and it was weird and surprising.*

Not all experiences were negative, we found a range of experiences from severely adverse events (around two-thirds of the participating girls) to calm, smooth transitions. The girls who felt prepared for menarche – regardless of the source of their information and preparedness – were more comfortable discussing menstruation in the present. These girls were more confident talking about a relaxed, uncomplicated experience of menarche, saying that they were “feeling the same”, that it was “nothing”, or “no fear or anxiety”. This improved menstrual experience was especially apparent among the older menarchal ages (15–16 years old) and for participants who stayed in school through eighth grade (14 years old), highlighting the exposure to some menstrual health education.

## Discussion

Our main aim in this paper was to explore menstrual preparedness among adolescent girls living in longstanding Palestinian refugee camps in the West Bank of the oPt and Jordan. We found that girls are relatively unprepared, increasing with low age, and receive mainly hygienic information at the menarchal event from their mothers. Barriers to “adequate, timely age-appropriate information”^4^ include socio-cultural factors, such as secrecy and embarrassment, amplified by the camp structure. Furthermore, our findings indicated a link between inadequate preparedness and the experience of the first menstruation as a dramatic event, with mixed feelings of fear, anxiety, and shock. This is consistent with results from previous research, from both within and outside the region.^8,10,37^ Two studies from Jordan highlighted this connection in different ways: in the first, adolescent girls reported frightening stories about their first menstruation and being inadequately prepared.^33^ The second showed that 82% of participants were not sufficiently prepared for menstruation, and they scored lower on menstrual attitudes and practices than those who were adequately prepared.^34^ Similarly, in a multi-generations quantitative study, Mexican girls and women consistently tended to feel regretful about their first experience, reporting shock and fear as the most dominant feelings which were more pronounced among those inadequately prepared or informed about menarche.^38^ Our findings support the notion that adequate menstrual preparedness can facilitate smooth transitions and positive menstrual experiences.^39^

### Preparedness beyond WASH

Our study revealed variations in menstrual preparedness in terms of adequacy and information timeliness. As Koff and Reirdan indicated,^19^ adequate menstrual preparedness should include information on menstrual biology and physical changes, practical aspects of hygiene and hygienic practices, and emotional and psychological support. Many of the menstrual health interventions in LMICs and refugee camps are dominated by WASH and MHM programmes, promoting solutions to meet the hygienic menstrual needs of adolescent girls, such as access to clean water, soap, and sanitary products as well as adequate privacy.^40^ In contrast to the many studies from LMICs that report inadequate access to toilet facilities and disposable pads, such as in India, Ghana, Kenya, Tanzania, and others,^2,8,9,25,40,41^ we found that the practical and hygienic preparedness for adolescent girls in the camps studied was adequate. Their preparedness was not hindered greatly by material limitations; disposable sanitary pads were easily available, and they had access to toilets with running water in their close-by homes.

Hence, while access to water, sanitation, and hygiene is less of a challenge in this setting, we found that there is a need for adequate information, including information about the biological transitions and non-secretive information that can provide emotional and psychological support. We found that information is also provided too late, leaving girls to suffer from traumatic menarchal events.

### Divergent notions of timeliness

We did not find much disagreement on whether information about menstrual preparedness has value for adolescent girls or not. However, we found that this information was incomplete and that the notions of timeliness of information provision varied, especially between the girls and their mothers. Most of the girls were informed about menstruation, primarily by their mothers and teachers; however, this happened after they reached menarche. Mothers seemed to communicate information too late in the pubertal period (after menarche), and often it tended to be incomplete, which is consistent with previous studies.^16,38,42,43^ The association of menstruation and sexuality is one of the reasons mothers do not talk about menstruation with their daughters early enough, as they fear that a conversation about menstruation can lead to age-inappropriate questions about sex and reproduction.^42^

Furthermore, the delay in the emotional preparedness for menarche was a big challenge for the participating girls. Although mothers tended to reassure their daughters and provide emotional support when they got their first period, the dramatic event had already happened. Menstrual health research has shown that girls and women who experienced dramatic menstrual first events tended to treat menstruation as a problem – as something they need to deal with and not to live with – that is, as something to control rather than a normal biological process.^22,44^ Adequate and timely menstrual preparation is crucial, as evident in previous studies^8,9,33,37^; hence, developing a shared understanding of timeliness is essential.

### Menstrual taboo *(Aib)*

This study showed that menstruation was a sensitive and silenced issue expressed through the concept of *aib*. We found *aib* to be overarching in all aspects of the menstrual experience, starting from the pre-menarchal phase and extending throughout girls’ and women’s lives across generations, silencing menstrual experiences and knowledge transfers. Hawkey et al. describe “cycles of shame”,^2^ in which women engage in negative constructions of menstruation, positioning it as shameful and something to be concealed. As Bobel writes about menstruation: “With notable exceptions, across cultures and historical eras, we socialise this biological process – including a serious inquiry into its forms, functions, and meaning – into hiding”.^44^ Examples of silencing menstruation are also found in non-camp settings in the region. In an Egyptian quantitative study on adolescent girls in secondary schools, for instance, only 50% responded to the survey because they believed it should not be addressed in public.^45^ Menstruation as *aib* is fundamentally based on its association with sexuality and the gender ideologies and patriarchy dominating Arab communities, including the refugee camps in this study. Female sexuality and reproduction are closely controlled, and discussions about anything associated with sexuality within families and at schools, especially for girls, is believed to potentially promote premarital sex, which is strictly prohibited in the camps and the region more broadly.^46^ Gender roles in a broad sense and the available social positions of women also inform the positionality of mothers in providing adequate information about menstrual health to their daughters. Although mothers were not targeted in this study, the girls’ statements explicitly point to the need to educate mothers about menarche and menstrual health and to work to empower women to break the silence around these topics.^2^ Many studies have revealed mothers’ limited role in educating their daughters; even if they are aware of its necessity, many mothers feel they lack the skills to do so.^43^

Moreover, as we found in the refugee camps, the socio-cultural norms, including *aib*, control of sexuality and gender roles and relations, inform discussions about menstruation across generations and also across professions and social insitutions and thus need to be addressed to achieve changes. Information about sensitive topics related to adolescents’ reproductive health is negotiated in refugee camps between the education sector and the family, excluding the health sector.^5^ Yet, the school curricula concerning menstrual health are often weak,^5^ particularly in conservative communities, such as Palestinian refugee camps.^47^ This was shown in a Jordan study where the school policies and practices did not allow for discussions of sexual and reproductive health topics directly or indirectly.^34^

The lack of privacy is also a big challenge for girls living with minimal indoor and outdoor spaces. Life within an extended family framework dominated by gender and patriarchal relationships and a conservative community implies that girls are keenly watched and observed.^28^ The environment of the camps, being both socially close-knit and crowded, enforces the social control of girls, not only their movements but also their conversations. Hence, the camp structure restrains open information flow on sensitive issues.^48^

### Menstrual preparedness and human rights: towards Comprehensive, Contextually appropriate, Timely Menstrual Preparedness (CCTMP) programmes

Menstruation is fundamentally linked to human rights and dignity, namely rights to health, education, work, non-discrimination, gender equality, and the right to access clean water and sanitation.^49^ When women and girls lack access to clean washing facilities and safe and effective methods of menstrual hygiene management, in addition to teasing, isolation, and shame associated with menstruation, they cannot manage their menstruation with dignity.^14^ Menstruation can become a period of deprivation and stigma for women and girls due to gender inequality, poverty, and negative traditions and norms, all of which can jeopardise their enjoyment of basic human rights.^3^

More specifically, inadequate menstrual preparedness is linked to health and dignity as a human right.^14,50^ When girls are unaware of what is happening to their bodies, they are vulnerable to social and emotional stressors that may negatively impact their psychological well-being.^15^ Additionally, a lack of accurate information may affect their health-seeking behaviour, and they may be unaware of when medical assistance is required. This is exacerbated by the stigma and taboo associated with menstruation, which discourages them from taking the necessary actions.^14^ On the other hand, many girls worldwide are denied their fundamental human right to education due to a lack of proper WASH facilities. This can affect their menstrual preparedness status coming through school education.^15^ A multifaceted approach is required to ensure the fulfilment of these fundamental rights for adolescent girls.^51^

On the International Women’s Day in 2019, the UN Human Rights Council raised the continued problem of menstrual health being a taboo, writing: “Persistent harmful socio-cultural norms, stigma, misconceptions and taboos around menstruation, continue to lead to exclusion and discrimination of women and girls”.^52^ Menstrual health and preparedness need to be better addressed locally, regionally, and globally. Hennegan and colleagues have advocated for a global definition of menstrual health that is comprehensive and unified for better research, policy, and practice.^4^ This followed another important paper by Wilson et al., who called for the inclusion of menstrual health under the general sexual and reproductive health and rights umbrella.^3^ These calls are the first step in comprehensively addressing menstruation, ensuring girls’ basic rights to achieve good menstrual health. The results of our study in the refugee camps in the West Bank and Jordan are consistent with this call. We argue that better menstrual preparedness is an important way to improve menstrual health. We strongly advocate for Comprehensive, Contextually Relevant, Timely Menstrual Preparedness (CCTMP) policies and interventions that start by the fifth grade (around nine years of age) at the latest. These interventions should include the biological, physical, practical, emotional, and psychosocial aspects of menstrual preparedness.

Structural, institutional, and socio-cultural barriers should be considered when developing menstrual preparedness interventions, and the notion of *aib* should be actively addressed. We found that breaking the silence and taboo around menstruation is possible in certain settings. The focus-group discussions provided an opportunity and encouraged girls to talk about their experiences, leading to a sense of empowerment when listening to their peers’ stories. Investing in participatory research approaches and promoting peer-to-peer education has also been reported to be effective in previous relevant studies, especially during adolescence and on topics related to puberty and menstruation.^53^

Building relationships with girls, mothers, and teachers is also a prerequisite for trust, acceptance, and change. We found one interesting example of how knowledge can be shared in a two-directional way across generations when the adolescent girls brought newly gained knowledge about menstrual health from school back home and argued and discussed this with their mothers and grandmothers.

Achieving better menstrual health is not the responsibility of the adolescent girls and other women alone. A community approach is needed where different stakeholders can collaborate to support the girls.^9^ These stakeholders include the family, educational, and health institutions, community and community institutions, and most importantly, governmental bodies with political will and power.^4^ We suggest that such initiatives can be launched under the umbrella of the educational system in the refugee camps, where the schools have been found to be the most reliable, acceptable, and safe space to facilitate systematic discussions about menstrual health. This would also provide all girls with a chance to learn, given that basic education is mandatory.^26^ Investing in private spaces where girls can discuss sensitive issues freely, without feeling shame, is essential. Given the refugee camp structure, schools or clinics can be appropriate options for gatherings. However, political will and commitment to providing spaces within these institutions are needed. Hence, this cannot be achieved without family and community acceptance and support. Mobilising the community to advocate for menstrual health as a priority to be addressed without shame or taboo would address the *aib* barrier. This could, for example, be accomplished by targeting community leaders and explaining the negative consequences of such control on girls’ health. A similar point was documented in a recent systematic review, emphasising the need for multidisciplinary action to meet the girls’ basic human rights of menstrual health and hygiene.^51^

**Figure 2.**
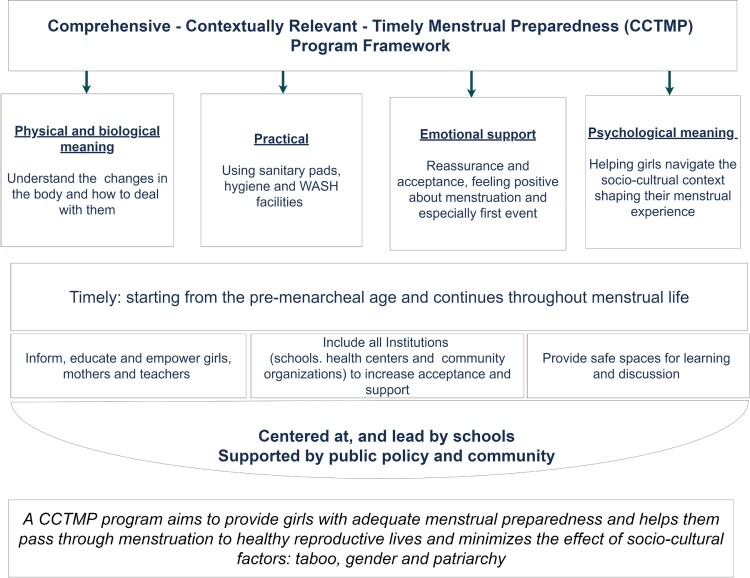
Comprehensive, Contextually Relevant, Timely Menstrual Preparedness Program Framework (CCTMP)

The following figure represents and sums up a general suggested framework for a comprehensive, contextually relevant, timely menstrual preparedness programme considering all aspects discussed in this paper (Figure 2).

### Strengths and limitations

To our knowledge, this topic has not been previously discussed in depth in the study locations. The study’s reliance on a significant amount of qualitative data to enhance understanding of the menstrual experience of adolescent girls in this particular environment is a major strength. In addition, the researchers who conducted the interviews had a solid grasp of the local community and knew how to approach individuals. Limitations of the study are the age limits of the participants and the lack of mothers in the sample. Given the menstrual experiences reported by girls, we see now that including girls younger than 15 years old would be a strength, and we strongly propose that the early adolescence period be included in future studies on menstrual education. Moreover, including mothers would also have strengthened our understanding of the intergenerational gap and modes of knowledge transition.

## Conclusion

Menstrual preparedness should be addressed beyond practicality and hygiene to include comprehensive and timely information shared in a way that can empower adolescent girls and other women, including mothers, grandmothers, teachers, health workers, and other professionals. Appropriate information can help girls and women appreciate their bodies and feel more positive about bodily functions and changes, acknowledging both biological and psychological needs. A collaborative approach with the participation of families, schools, health services, and local community organisations is encouraged to provide girls with adequate, timely, comprehensive menstrual preparedness that allows for a healthy, comfortable transition to adulthood and a healthy reproductive life. This can only be done by placing menarche firmly within the ambit of sexual and reproductive health services and by intersectoral collaboration, investing in mothers’ and adolescent girls’ education, and empowering school teachers and counsellors to carry out their roles effectively.

## Supplementary Material

Appendix 1: Summary of Focus Group Discussions (FGDs) and In-Depth-Interviews (IDIs) girls’ characteristics.

Appendix 2: The experience of menarche and menstruation among adolescent girl camp dwellers in Palestine (West Bank) and Jordan.

## Footnotes

* Although the study targeted girls 15–18 years old, a few girls 14 years old or older than 18 years (up to 21) were invited by the local networks to participate. Their input was included in the analysis because we believe that the research topic would not be affected by this minor age difference, and this was clear from their input.

## References

[1] Bobel C. The managed body: developing girls and menstrual health in the global south. Cham: Springer International Publishing: Imprint: Palgrave Macmillan; 2019.

[2] Hawkey AJ, Ussher JM, Perz J, et al. Experiences and constructions of menarche and menstruation among migrant and refugee women. Qual Health Res. 2017;27(10):1473–1490. 27742765 10.1177/1049732316672639

[3] Wilson LC, Rademacher KH, Rosenbaum J, et al. Seeking synergies: understanding the evidence that links menstrual health and sexual and reproductive health and rights. Sex Reprod Health Matters. 2021;29(1):1882791. 33599162 10.1080/26410397.2021.1882791 PMC8009024

[4] Hennegan J, Winkler IT, Bobel C, et al. Menstrual health: a definition for policy, practice, and research. Sex Reprod Health Matters. 2021;29(1):1911618. 33910492 10.1080/26410397.2021.1911618 PMC8098749

[5] Sommer M, Cherenack E, Blake S, et al., editors. WASH in schools: empowers girls’ education. Menstrual Hygiene Management in Schools Virtual Conference 2014. New York: United Nations Children’s Fund and Columbia University; 2015.

[6] Orringer K, Gahagan S. Adolescent girls define menstruation: a multiethnic exploratory study. Health Care Women Int. 2010;31(9):831–847. 20677040 10.1080/07399331003653782

[7] Hennegan J, Shannon AK, Rubli J, et al. Women’s and girls’ experiences of menstruation in low- and middle-income countries: a systematic review and qualitative metasynthesis. PLoS Med. 2019;16(5):e1002803. 31095568 10.1371/journal.pmed.1002803 PMC6521998

[8] Chandra-Mouli V, Patel SV. Mapping the knowledge and understanding of menarche, menstrual hygiene and menstrual health among adolescent girls in low- and middle-income countries. Reprod Health. 2017;14:30. 28249610 10.1186/s12978-017-0293-6 PMC5333382

[9] Coast E, Lattof SR, Strong J. Puberty and menstruation knowledge among young adolescents in low- and middle-income countries: a scoping review. Int J Public Health. 2019;64(2):293–304. 30740629 10.1007/s00038-019-01209-0 PMC6439145

[10] Carol C, Beausang AGR. Young western women’s experiences of menarche and menstruation. Health Care Women Int. 2000;21(6):517–528. 11235283 10.1080/07399330050130304

[11] Moffat N, Pickering L. “Out of order”: The double burden of menstrual etiquette and the subtle exclusion of women from public space in Scotland. Sociol Rev. 2019;67(4):766–787.

[12] Dars S, Sayed K, Yousufzai Z. Relationship of menstrual irregularities to BMI and nutritional status in adolescent girls. Pak J Med Sci. 2014;30(1):141–144. 24639848 10.12669/pjms.301.3949 PMC3955559

[13] Starrs AM, Ezeh AC, Barker G, et al. Accelerate progress – sexual and reproductive health and rights for all: report of the Guttmacher–Lancet Commission. The Lancet. 2018;391(10140):2642–2692. 10.1016/S0140-6736(18)30293-9 29753597

[14] Babbar K, Martin J, Ruiz J, et al. Menstrual health is a public health and human rights issue. The Lancet Public Health. 2022;7(1):e10–ee1. 34717798 10.1016/S2468-2667(21)00212-7 PMC8552814

[15] Human Right Watch. Understanding menstrual hygiene management and human rights. Report. US: Human Rights Watch; 2017.

[16] Chang Y-T, Hayter M, Wu S-C. A systematic review and meta-ethnography of the qualitative literature: experiences of the menarche. J Clin Nurs. 2010;19(3–4):447–460. 20500285 10.1111/j.1365-2702.2009.03019.x

[17] Sommer M, Sutherland C, Chandra-Mouli V. Putting menarche and girls into the global population health agenda. Reprod Health. 2015;12:24. 25889785 10.1186/s12978-015-0009-8 PMC4396832

[18] Greif EB, Ulman KJ. The psychological impact of menarche on early adolescent females: a review of the literature. Child Dev. 1982;53(6):1413. 6756807

[19] Koff E, Rierdan J. Preparing girls for menstruation: recommendations from adolescent girls. Adolescence. 1995;30(120):795–811. 8588517

[20] Tull K. Period poverty impact on the economic empowerment of women. Brighton: Institute of Development Studies; 2019 (K4D Helpdesk Report 536).

[21] Medina-Perucha L, Jacques-Aviñó C, Valls-Llobet C, et al. Menstrual health and period poverty among young people who menstruate in the Barcelona metropolitan area (Spain): protocol of a mixed-methods study. Br Med J Open. 2020;10(7):e035914. 10.1136/bmjopen-2019-035914 PMC7394147 32727738

[22] Crichton J, Okal J, Kabiru CW, et al. Emotional and psychosocial aspects of menstrual poverty in resource-poor settings: a qualitative study of the experiences of adolescent girls in an informal settlement in Nairobi. Health Care Women Int. 2013;34(10):891–916. 23570366 10.1080/07399332.2012.740112

[23] Sommer M, Ackatia-Armah N, Connolly S, et al. A comparison of the menstruation and education experiences of girls in Tanzania, Ghana, Cambodia and Ethiopia. Compare: A J Comp Int Educ. 2015;45(4):589–609.

[24] Sommer M, Schmitt ML, Clatworthy D, et al. What is the scope for addressing menstrual hygiene management in complex humanitarian emergencies? A global review. Waterlines. 2016;35(3):245–264.

[25] Schmitt M, Clatworthy D, Ratnayake R, et al. Understanding the menstrual hygiene management challenges facing displaced girls and women: findings from qualitative assessments in Myanmar and Lebanon. Confl Health. 2017;11(1):1–11. DOI:10.1186/s13031-017-0121-1 29046714 PMC5641996

[26] UNRWA. Palestine Refugees [cited 2017 17 December]. Available from: https://www.unrwa.org/palestine-refugees

[27] Feldman I. What is a camp? Legitimate refugee lives in spaces of long-term displacement. Geoforum. 2015;66:244–252.

[28] Hanafi S. Palestinian refugee camps: disciplinary space and territory of exception, CARIM AS 2008/44. Robert Schuman Center for Advanced Studies, San Dominico di Fiesole (FI): European University Institute; 2008.

[29] On the occasion of the international day of refugees (20/06/2016). [press release]. Ramallah, Palestine: Palestinian Central Bureau of Statistics; 2016.

[30] Abu-Rmeileh NME, Ghandour R, Tucktuck M, et al. Research priority-setting: reproductive health in the occupied Palestinian territory. Reprod Health. 2018;15(1):27. 29433508 10.1186/s12978-018-0472-0 PMC5810115

[31] Shalash A, Alsalman HM, Hamed A, et al. The range and nature of reproductive health research in the occupied Palestinian territory: a scoping review. Reprod Health. 2019;16(1):41. 30944010 10.1186/s12978-019-0699-4 PMC6448219

[32] Shalabi-Abbas E, Dweikat S, Al Gazawy I, et al. Knowledge and self-care practices in adolescent girls living in Nablus district during menstruation: a cross-sectional study [conference paper]. The Lancet 2018/02/21/2018. p. S10. 10.1016/S0140-6736(18)30376-3 29553407

[33] Al Omari O, Abdel Razeq NM, Fooladi MM. Experience of menarche among Jordanian adolescent girls: an interpretive phenomenological analysis. J Pediatric Adolescent Gynecolog. 2016;29(3):246–251. 10.1016/j.jpag.2015.09.005 26463575

[34] Jarrah SS, Kamel AA. Attitudes and practices of school-aged girls towards menstruation. Int J Nurs Pract. 2012;18(3):308–315. 22621303 10.1111/j.1440-172X.2012.02032.x

[35] ICPH. Reproductive health needs of Palestinian refugee camp adolescent girls: from evidence to policy-project description: Institute of Community and Public Health, Palestine; 2018. Available from: http://icph.birzeit.edu/research/projects/reproductive-health-needs-palestinian-refugee-camp-adolescent-girls-evidence

[36] Clarke V, Braun V. Thematic analysis. New York: Springer; 2014. p. 1947–1952.

[37] Eswi A, Helal H, Elarousy WJJAS. Menstrual attitude and knowledge among Egyptian female adolescents. The J Am Sci. 2012;8(6):555–565.

[38] Marván ML, Morales C, Cortés-Iniestra S. Emotional reactions to menarche among Mexican women of different generations. Sex Roles. 2006;54(5):323–330.

[39] Sarkar I, Dobe M, Dasgupta A, et al. Determinants of menstrual hygiene among school going adolescent girls in a rural area of West Bengal. J Family Med Prim Care. 2017;6(3):583–588. 29417013 10.4103/2249-4863.222054 PMC5787960

[40] VanLeeuwen C, Torondel B. Improving menstrual hygiene management in emergency contexts: literature review of current perspectives. Int J Womens Health. 2018;10:169–186. 29692636 10.2147/IJWH.S135587 PMC5901152

[41] Sommer M. Menstrual hygiene management in humanitarian emergencies: gaps and recommendations. Waterlines. 2012;31(1/2):83–104.

[42] Garg S, Sharma N, Sahay R. Socio-cultural aspects of menstruation in an urban slum in Delhi, India. Reprod Health Matters. 2001;9(17):16–25. 11468832 10.1016/s0968-8080(01)90004-7

[43] Sooki Z, Shariati M, Chaman R, et al. The role of mother in informing girls about puberty: a meta-analysis study. Nurs Midwifery Stud. 2016;5(1):e30360–e. 27331056 10.17795/nmsjournal30360 PMC4915208

[44] Bobel C, Winkler IT, Fahs B, et al. The palgrave handbook of critical menstruation studies. Singapore: Springer Singapore: Imprint: Palgrave Macmillan; 2020. 33347099

[45] Abdelmoty HI, Youssef MA, Abdallah S, et al. Menstrual patterns and disorders among secondary school adolescents in Egypt. A cross-sectional survey. BMC Womens Health. 2015;15:70. 26341264 10.1186/s12905-015-0228-8 PMC4560881

[46] DeJong J, El-Khoury G. Reproductive health of Arab young people. Br Med J. 2006;333(7573):849–851. 17053245 10.1136/bmj.38993.460197.68 PMC1618449

[47] Helwani L. Reframing the bloody hell: menstrual rituals and practices among Arab and Arab-Americans [Master thesis]: Harvard Extension School; 2016.

[48] Marshall DJ. “We have a place to play, but someone else controls it”: girls’ mobility and access to space in a Palestinian refugee camp. Global Stud Childhood. 2015;5(2):191–205.

[49] UNFPA. Menstruation and human rights - Frequently asked questions 2021 [cited 2022 March 10]. Available from: https://www.unfpa.org/menstruationfaq#menstruation%20and%20human%20rights

[50] Carneiro MM. Menstrual poverty: enough is enough. Women Health. 2021;61(8):721–722. 34517781 10.1080/03630242.2021.1970502

[51] Sood S, Stevens S, Okumura M, et al. A systematic review of menstrual health and hygiene management (MHHM) as a human right for adolescents girls. Int J Sex Health. 2022:1–20. DOI:10.1080/19317611.2022.2050874 PMC10903676 38596276

[52] United Nations Human Rights. International women’s day, 8 March 2019. Available from: https://www.ohchr.org/en/press-releases/2019/03/international-womens-day-8-march-2019womens-menstrual-health-should-no

[53] Knibbs S, Price N. Peer education in sexual and reproductive health programming: a Cambodian case study. Dev Pract. 2009;19(1):39–50.

