# Supplementary material for: Coming of age: a qualitative study of adolescent girls’ menstrual preparedness in Palestinian refugee camps in the West Bank and Jordan
Source: Sex Reprod Health Matters. 2022 Sep 21;30(1):2111793. doi: 10.1080/26410397.2022.2111793 (PMC9518256; doi:10.1080/26410397.2022.2111793)
Supplement: Appendix 1: Summary of Focus Group Discussions (FGDs) and In-Depth-Interviews (IDIs) girls’ characteristics. [file ZRHM_A_2111793_SM0520.pdf]

Appendix 1: Summary of Focus Group Discussions (FGDs) and In-Depth-Interviews (IDIs) girls' characteristics

	West Bank	Jordan	Total
FGDs			
Number of FGD	12	11	23
Number of participating girls in all FGDs	114	79	193
Age	14-19 years old	14-19 years old<br>(4 >19)	14-19 years old<br>(4 >19)
Enrollment status	4 completed high school<br>100 currently enrolled at school	34 dropped out before completing high school<br>45 currently enrolled at school	145 Currently enrolled at school<br>4 completed high school<br>34 dropped out before completing high school
Marital status	all never married	2 separated<br>2 divorced<br>75 never married	2 separated<br>2 divorced<br>189 never married
IDIs			
Number of IDI	22	17	39
Age	14-19<br>(2 >19)	14-19	14-19<br>(2 >19)
Enrollment status			
Marital status	17 Never married<br>4 currently married<br>1 separated	16 Never married<br>1 currently married	33 Never married<br>5 currently married<br>1 separated
