# Supplementary material for: Coming of age: a qualitative study of adolescent girls’ menstrual preparedness in Palestinian refugee camps in the West Bank and Jordan
Source: Sex Reprod Health Matters. 2022 Sep 21;30(1):2111793. doi: 10.1080/26410397.2022.2111793 (PMC9518256; doi:10.1080/26410397.2022.2111793)
Supplement: Appendix 2: The experience of menarche and menstruation among adolescent girl camp dwellers in Palestine (West Bank) and Jordan. [file ZRHM_A_2111793_SM0514.pdf]

Appendix 2:

The experience of menarche and menstruation among adolescent girl camp dwellers in Palestine (West Bank) and Jordan

In-depth interview guide with girls 15-18 years old :

Aim: to provide description of behaviors / experience / knowledge/ attitudes and values related to menarche and menstruation

Identification: *Name (can be fictitious name), Age? Housing: Inside or outside the camp? Are you enrolled to school, (if no, why and what grade did you finish?)*

Experience of menarche and menstruation

Experience of menstruation

Probing to reach the menstruation issue: Do you feel any changes in appetite or other things during certain times of the month? Are these changes related to the date of the menstrual cycle?

- Tell me about your menstrual cycle
  - Are there any changes you experience before or during your period? *Probing: pain, changes in weight and appetite, psychological changes, etc.*
  - What do you do to cope with these changes?
  - Do you suffer from any problems/ disturbance related to menstrual cycle? Have you heard about any? What causes these problems? How can it be solved?
  - Do you feel any pain during your period, how do you deal with it, take medications, home remedies, etc. Do you miss school because of this pain?
- How to keep your personal hygiene?
  - *Probing : during the period: daily sanitary towels, hair removal, showers, changing underwear ...), and during the month*
- Do you know when you will have your period?

Experience of menarche

- Tell me about your experience with the first time you had your period? How old were you? And how did you deal with it?

  Probing
  - Did you know anything about it before its onset? Why it happens? How long it lasts ?
  - What were your sources of information
- What are the bodily/ physical changes you've experienced since you've started menstruating?

- When you’ve started menstruating, have you changed the way you see or deal with yourself? Have you noticed any changes in the way others see or deal with you (your family, your friends, at school, the community ...)?

Needs in terms of knowledge and services

- What topics/ conditions (related to menarche and menstruation) you feel you need to know more about?
- When you need such knowledge, where do you usually go? Or whom do you consult?
- In your opinion, what types of health services related to these conditions are available and what are missing.

Focus group guide:

Aim: to understand the social construction of meaning and knowledge (social norms and values) related to menarche and menstruation.

Identification information

- What are your ages?
- Are you enrolled (school, college, work...)
    - If enrolled to school: which grades are you?
    - *Those are mainly 9th grade in the West Bank and 10th grade in Jordan (UNRWA schools provides education only to these classes, then girls has to go outside camps to join governmental or other schools)*
- Do you live inside or outside the camp/ have you ever lived in the camp?
- Any of you are engaged/ married/ other …

- Menstruation:
    - We will talk a little bit about your menstrual cycle? Do you know why do you have your menstrual cycle/ period?
    - How often do you get your menstrual cycle? Do you know when you will get it?
    - From where do you get information about your menstruation
        - Does the school have any role?
    - How do you evaluate your life during menstruation?
        - How do you feel? Are there any changes you feel during or before its onset (both physical and psychological)? (weight, appetite, psychological feelings, pain...)
        - Do you skip school because of menstruation/ menstrual pain?
        - Will menstruation affect your daily activities?

- For those who have menstrual pain, how do you deal with it? Home remedies, medications, other …
- How do you care for your personal hygiene during menstruation ? Do you use toilets at schools?
- Have you ever heard about any problems related to menstruation? Something you faced, your friends, relatives, … explore

- Menarche:

  Now we need to talk about your first time getting menstruation? Do you remember? Talk to us about your experience?

  - Did you know anything about it before you got it?
  - How old were you?
  - How did you deal with the situation? Who did you talk to?
  - Do you remember any bodily changes that followed your menarche?
  - When you reached menarche, did notice any changes in the way people look at you (your family, relatives, neighbors,…)
  - Did you change the way you look at your self? Describe
